# Atheroprotective Vaccination with MHC-II Restricted Peptides from ApoB-100

**DOI:** 10.3389/fimmu.2013.00493

**Published:** 2013-12-27

**Authors:** Kevin Tse, Ayelet Gonen, John Sidney, Hui Ouyang, Joseph L. Witztum, Alessandro Sette, Harley Tse, Klaus Ley

**Affiliations:** ^1^Department of Medicine, Division of Rheumatology, Allergy and Immunology, University of California at San Diego, La Jolla, CA, USA; ^2^Division of Inflammation Biology, La Jolla Institute for Allergy and Immunology, La Jolla, CA, USA; ^3^Department of Medicine, Division of Endocrinology and Metabolism, University of California at San Diego, La Jolla, CA, USA; ^4^Division of Vaccine Discovery, La Jolla Institute for Allergy and Immunology, La Jolla, CA, USA; ^5^Department of Immunology and Microbiology, School of Medicine, Wayne State University, Detroit, MI, USA

**Keywords:** T-cell, atherosclerosis, vaccination, inflammation

## Abstract

**Background:** Subsets of CD4^+^ T-cells have been proposed to serve differential roles in the development of atherosclerosis. Some T-cell types are atherogenic (T-helper type 1), while others are thought to be protective (regulatory T-cells). Lineage commitment toward one type of helper T-cell versus another is strongly influenced by the inflammatory context in which antigens are recognized. Immunization of atherosclerosis-prone mice with low-density lipoprotein (LDL) or its oxidized derivative (ox-LDL) is known to be atheroprotective. However, the antigen specificity of the T-cells induced by vaccination and the mechanism of protection are not known.

**Methods:** Identification of two peptide fragments (ApoB_3501–3516_ and ApoB_978–993_) from murine ApoB-100 was facilitated using I-Ab prediction models, and their binding to I-Ab determined. Utilizing a vaccination scheme based on complete and incomplete Freund’s adjuvant (CFA and IFA) [1 × CFA + 4 × IFA], we immunized Apoe^−/−^mice with ApoB_3501–3516_ or ApoB_978–993_ emulsified in CFA once and subsequently boosted in IFA four times over 15 weeks. Spleens, lymph nodes, and aortas were harvested and evaluated by flow cytometry and real time RT-PCR. Total atherosclerotic plaque burden was determined by aortic pinning and by aortic root histology.

**Results:** Mice immunized with ApoB_3501–3516_ or ApoB_978–993_ demonstrated 40% reduction in overall plaque burden when compared to adjuvant-only control mice. Aortic root frozen sections from ApoB_3501–3516_ immunized mice showed a >60% reduction in aortic sinus plaque development. Aortas from both ApoB_3501–3516_ and ApoB_978–993_ immunized mice contained significantly more mRNA for IL-10. Both antigen-specific IgG1 and IgG2c titers were elevated in ApoB_3501–3516_ or ApoB_978–993_ immunized mice, suggesting helper T-cell immune activity after immunization.

**Conclusion:** Our data show that MHC Class II restricted ApoB-100 peptides can be atheroprotective, potentially through a mechanism involving elevated IL-10.

## Introduction

Improvements in the treatment and prevention of cardiovascular disease (CVD) resulted in a 30% reduction in its mortality rate between 1998 and 2008. Despite this, a recent summary statement from the American Heart Association estimates that each year 785,000 Americans will have a new heart attack, and that someone dies from a coronary event every 60 s (1). These statistics underscore the importance of research that seeks to develop novel therapies in the fight against atherosclerosis.

Recent discoveries suggest that local arterial tissue inflammation is likely a major instigator in the development of atherosclerosis (2–6). This inflammation is mediated, in part, by local immunologic processes at the site of plaque lesions which involve both innate and adaptive immunity (2, 6–10). The antigens in atherosclerosis driving both pro-atherosclerotic and atheroprotective immune responses are not known with certainty. Oxidized low-density lipoprotein (LDL) (ox-LDL), and the lipoprotein portion of LDL (ApoB-100) have been proposed as candidate antigens (10–13). Recent evidence suggests that autoantigens are presented to antigen-experienced CD4^+^ T-cells by antigen presenting cells (APCs) residing in the atherosclerotic arteries (14). All T-cell subsets (CD4^+^, CD8^+^, TCRγδ^+^, NKT-cells) have been found in mouse and human plaques (2, 5, 15, 16). In particular, the presence of CD4^+^FoxP3^+^ regulatory T-cells (T_REGS_) in these plaques (17–19) indicates that inflammation within the plaque is a regulated process, offering hope that therapeutics can be designed targeting T-cell function and differentiation.

Regulatory T-cells are the focus of many studies in atherosclerosis because of their atheroprotective potential. T_REGS_ are reduced in atherosclerotic plaques (18) and in circulating peripheral blood of human subjects with CVD (20) compared to healthy subjects. In murine studies, adoptive transfer of a subset of T_REGS_ (Tr1 cells, CD4^+^FoxP3^±^IL-10^+^) administered to Apoe^−/−^mice showed a significant decrease in pro-atherosclerotic IFN-γ production, increased IL-10 production, and significant reduction in atherosclerotic lesion size when compared with control mice (21). IL-10, secreted by T_REGS_, is atheroprotective and protects both from fatty streak formation and atherosclerotic plaque formation (15, 22).

Several studies have reported a protective effect of vaccination with LDL, or ox-LDL, on the development of atherosclerotic plaque (23–25). However, the mechanism by which these immunizations convey atheroprotection is an ongoing area of research. One line of investigation suggests that protective autoantibodies generated during immunization with ox-LDL might be the source of atheroprotection (24, 26, 27). This was initially a promising hypothesis which subsequently led to the search for atheroprotective B-cell (antibody) epitopes. The discovery of such epitopes was first reported by Fredrikson et al. (28) in 2003. These researchers assessed the binding of endogenous antibodies (from pooled human plasma in a case-control cohort of patients with a history of acute coronary heart events) and identified several epitope sequences from human ApoB-100 that bound to endogenous human antibodies. Since then several of these peptides have been used to vaccinate mice, including P2 (TRFKHLRKYTYNYEAESSS) (29), P143 (IALDDAKINFNEKLSQLQTY) (30), and P210 (KTTKQSFDLSVKAQYKKNKH) (30) each conferring ~40–60% atheroprotection. While the original premise of immunizing with B-cell epitopes was atheroprotection via an increase in peptide-specific antibody levels, this was eventually determined to not be the case (31).

More recently, researchers have sought to describe the changes in cellular (rather than humoral) immunity that may result from immunization with B-cell epitopes from ApoB-100. Several recent papers have reported an increase in FoxP3^+^ expressing T_REGS_ in secondary lymphoid organs [spleens (32) and lymph nodes (33)] after immunization of mice with P210. While these findings are consistent with reports that suggest T_REGS_ might be protective in atherosclerosis (17–19), it is unclear how T-cells can be directly affected by vaccination with peptide sequences originally recognized as B-cell epitopes. This is especially peculiar in the case of P210, which has been shown not to bind to I-Ab (the MHC Class II allele expressed by both Apoe^−/−^ and LDL-R^−/−^ mice) (34), which is a prerequisite for any direct interaction with T-cell receptors. Subsequent studies have further described the changes to the T-cell compartment after immunization with B-cell epitopes (in particular the CD4^+^CD25^+^FoxP3^+^ T_REG_ population) (35). However, there are currently no T-cell epitopes related to atherosclerosis that have been described. Knowledge of such epitopes would help to better characterize T-cell phenotypes after peptide immunization in the treatment of atherosclerosis.

The existence of atherosclerosis-related CD4^+^ T-cell epitopes is suggested by several experiments. First, human CD4^+^ T-cell clones derived from atherosclerotic plaques responded to incubation with ox-LDL and autologous monocytes (acting as APCs) by proliferation and production of cytokine (IFNγ) in an antigen-specific, HLA-DR-restricted manner (11). More recently, live-cell imaging of explanted aortas from CD11cYFPApoe^−/−^ mice after 12 weeks of western diet (WD) showed that activated CD44^hi^CD62L^lo^ Apoe^−/−^ CD4^+^ T-cells isolated from Apoe^−/−^ mice, but not wild-type mice, productively interacted with CD11c-YFP^+^ APCs in the aortic wall (14). These T-cells were effector-memory CD4^+^ T-cells, had long interactions with APCs in the vessel wall and slowed migration speeds compared to T-cells isolated from naïve wild-type C57BL/6 mice. Furthermore, the “productiveness” of these interactions was demonstrated by increased T-cell proliferation and induction of the pro-inflammatory T_H_1 cytokines (IFNγ and TNF). These data suggest that endogenous T-cell antigens are presented in the aortic wall. It is therefore reasonable to suspect that these interactions may be promoting, or inhibiting, atherosclerosis by driving effector T-cell or regulatory T-cell responses, respectively. Manipulation of either these antigens or the T-cells that respond to these antigens would be of great therapeutic value.

Here, we report the discovery of two novel MHC-II restricted peptides identified in the murine ApoB-100 molecule. These peptides have high affinity for I-A^b^ (the MHC class II molecule in C57BL/6 mice), and injection of these two peptides into Apoe^−/−^ mice in complete Freund’s adjuvant (CFA), followed by four boosts in incomplete Freund’s adjuvant (IFA), reduces atherosclerosis. These data suggest that atheroprotective CD4^+^ T-cell vaccines can be developed.

## Materials and Methods

### Mice

Eight-week-old female *Apoe*^−/−^ mice on C57BL/6 background were purchased from Jackson Laboratories (Bar Harbor, ME, USA). Mice were housed in a specific pathogen free environment and fed chow diet until 10 weeks of age. At 10 weeks of age, mice were started on WD (adjusted calories diet with 42% from fat, Harlan Labs Cat #: TD.88137, CA, USA) and remained on WD until sacrifice at 23 weeks old.

### Peptides

Using previously described algorithms (36–38) for predicting I-A^b^ (MHC Class II) peptide binding motifs in a protein molecule, we scanned the entire 4505 amino acid sequence of murine ApoB-100 and identified candidate sequences based solely on matches for I-A^b^ binding. Six of these were selected based on favorable anchor residue and hydrophobicity profiles. These sequences are listed in Table 1. Although the minimal sequence to bind to the MHC Class II peptide groove is a 9-mer, all peptides were custom synthesized (Genemed Synthesis, Inc., San Antonio, TX, USA) as 15-mers to build “ends” on to the peptides for better binding. Peptides were synthesized in 0.1% trifluoroacetic acid (TFA) for sterility.

**Table 1:** **Peptide sequences and I-A^b^ binding affinity as determined by IC50**.

Peptide sequences	Mouse ApoB-100 position	H-2 I-A^b^ (IC50 nM)
NNYALFLSPRAQQAS	4505–4520	1295
SRATLYALSHAVNSY	438–453	1368
**SQEYSGSVANEANVY**	**3501–3516**	**4.3**
YENFAASNKLDVTFS	1578–1593	∞
HLEYVSSELRKSLQV	4054–4069	22176
**TGAYSNASSTESASY**	**978–993**	**7.3**
GWYRSPFSRVVHLY	MOG_38–51_	354
ISQAVHAAHAEINE	OVA_324–337_	400

*MOG, *Mus musculus* myelin oligodendrocyte glycoprotein; OVA, *Gallus gallus* ovalbumin. Bold font indicates ApoB_3501–3516_ and ApoB_978–993_*.

### I-A^b^ binding affinity assay

We measured I-A^b^ binding affinity using a classical competitive inhibition assay utilizing purified MHC and high affinity radio-labeled I-A^b^ ligands (39). Their ability to displace the radio-labeled I-A^b^ ligand was measured, and an inhibitory concentration required to displace 50% of the radio-labeled ligand (IC_50_) was determined. Under the conditions utilized, where (label) < (MHC) and IC50 ≥ (MHC), the measured IC50 values are reasonable approximations of the true *K*_d_ values.

### Antigen-specific proliferation

Peptides were emulsified in CFA and mice were immunized with 200 μg of peptide subcutaneously. Ten days later, draining lymph node cells were harvested and single cell suspensions were made. 5 × 10^5^ viable cells per microtiter well were cultured with 100 μg/mL of relevant (or irrelevant) peptide for 4 days. Purified protein derivative (PPD) served as a positive control. Sixteen hours before harvesting, 1 mCi of tritiated thymidine (^3^H) was added to each well. Cells were harvested and incorporation of ^3^H was determined in a scintillation counter. Results are expressed as stimulation index using the following formula:

$$\begin{array}{llll} & \mathrm{Stimulation}\;\mathrm{index}\;\left(\mathrm{SI}\right)\; & & \;\\ & \;=\frac{\mathrm{cpm}\;\mathrm{experimental}\;-\;\mathrm{cpm}\;\mathrm{media}\;\mathrm{control}}{\mathrm{cpm}\;\mathrm{media}\;\mathrm{control}}\; & & \; \end{array}$$

### Atheroprotective immunization

Previous work (25) has shown that atheroprotection through immunization could be achieved by injecting mice with LDL (or ox-LDL) with a combination of CFA initially, followed by antigen in IFA for booster immunizations. Using the same immunization model, 50 μg of ApoB_3501–3516_ or ApoB_978–993_ (diluted in PBS) was emulsified in equal volumes of CFA (BD Difco, Sparks, MD, USA) and injected into the subcutaneous inguinal area at 8 weeks of age. Repeated boosters with 25 μg of ApoB_3501–3516_ or ApoB_978–993_ emulsified in IFA (BD Difco, Sparks, MD, USA) were administered intraperitoneally at age 12, 16, 20, and 22 weeks. This immunization scheme will be referred to as 1× CFA + 4× IFA for the remainder of this manuscript. Mice were sacrificed at age 23 weeks and organs were harvested for analysis. Control immunizations with PBS emulsified in CFA and IFA were also performed. MOG_35–55_ [MEVGWYRSPFSRVVHLYRNGK, (40)] immunizations were also done under identical conditions with the same adjuvants.

### Atherosclerosis quantification

Aortic root sections were examined as follows. Hearts were harvested, placed in Optimal Cutting Temperature medium (OCT, Electron Microscopy Sciences, Hatfield, PA, USA), and frozen at −80°C. Beginning at the first appearance of the tri-leaflet aortic valve, successive 5 μm transverse sections were made for a distance of 100 μm. From these, we analyzed every other section, for a total of 10 sections per root. Sections were then stained with Oil Red O and counter-stained with hematoxylin. Extent of atherosclerosis was then determined as the area involved on each section. To measure en face lesion formation, the whole aorta was carefully cleaned *in situ* and then the whole aorta pinned out after paraformaldehyde incubation at RT for at least 2 h. Staining for atherosclerotic plaque was performed by incubating samples in Sudan IV. Quantification was performed using ImagePro software (Media Cybernetics, Rockville, MD, USA).

### Lipid analysis

Mouse whole blood was collected by cardiac heart puncture during organ harvest. No anticoagulant was used. The blood was placed on ice for at least 3–6 h, and then spun at 6300 rpm for 15 min at 4°C. The supernatant was collected and frozen at −80°C until analysis to reduce multiple freeze/thaw cycles. Individual samples were then analyzed by Roche COBAS 8000 Analyzer (Roche Diagnostics, Indianapolis, IN, USA).

### Measurement of antibody titers to ApoB_3501–3516_ and ApoB_978–993_

Antibody titers in plasma were determined by chemiluminescent enzyme immunoassay as previously described (41). In brief, white “U” bottom plates (Thermo Lab systems, USA) were coated with various antigens at 5 μg/mL in PBS for overnight incubation. Following blocking with 1% BSA-TBS serum was added in increasing dilutions and incubated at RT for 90 min. Bound antibodies levels were detected using appropriate alkaline phosphatase-conjugated secondary antibodies and a 50% aqueous solution of LumiPhos 530 (Lumigen, USA). Data are expressed as relative light units counted per 100 ms (RLU/100 ms).

### Flow cytometry

Aortas, lymph nodes, and spleens were digested as previously described (42). Aortic cell suspensions, spleens, and lymph nodes were individually pressed through a 70-μm filter and incubated for 30–60 min in complete RPMI to encourage CD4 re-expression. Approximately (1–2) × 10^6^ cells were then placed into 96-well round bottom plates and incubated for 5 min with Fc Block (1:200), and subsequently stained with primary antibody [CD45-PerCP 1:50 (BioLegend, San Diego, CA, USA, Cat#103130), CD4-PE-Cy7 1:50 (eBioscience, San Diego, CA, USA, Cat#25-0041-82), TCRβ-AF700 1:50 (BioLegend, Cat#109224), and Live/Dead Aqua 1:200 (Invitrogen, Grand Island, NY, USA, Cat# L34957)] for 45–60 min. Plates were washed twice and then incubated with Fix/Perm buffer solution (eBioscience, Cat# 00-5523-00) for additional 30 min. Plates were again washed twice with a permeabilization buffer prior to intracellular staining with intracellular transcription factor staining [FoxP3-efluor450 1:50 (eBioscience, Cat# 48-5773-82)]. Intracellular staining was performed for 30–45 min and plates were washed twice with permeabilization buffer solution. Samples were analyzed by LSR-II (BD Biosciences, San Jose, CA, USA). Data was acquired on FACSDiva software (BD Biosciences) and analyzed by FlowJo (Ashland, OH, USA).

### Quantitative RT-PCR

Spleens, lymph nodes, and aortas were placed in 50 μL of RNALater from Qiagen (Valencia, CA, USA) immediately after harvest. QIAShredder kit (Valencia, CA, USA) was used to homogenize each sample after Trizol treatment. RNA extraction performed using RNeasy Mini kit (Valencia, CA, USA). RNA was converted to cDNA using iScript Reverse Transcription kit for RT-qPCR (BioRad, Hercules, CA, USA). Primers were commercially obtained as part of a Taqman PCR kit (Life Technologies, New York, NY, USA), and included CD4, IFNγ, TNFα, IL-2, IL-4, IL-10, IL-17A, Tbx21 (Tbet), GATA3, RORγT, and FoxP3. Housekeeping genes used in the analysis were one of two ribosomal proteins, Rpl32 or Rpl13A.

### Statistical analysis

Between groups analysis was performed by one-way ANOVA. Data are expressed as mean ± SEM. *P-*values <0.05 were considered significant.

## Results

### Identifying candidate epitopes ApoB_3501–3516_ and ApoB_978–993_

A peptide can only be recognized by a CD4^+^ helper T-cell if it is bound to MHC class II. Because *Apoe*^−/−^ mice are on a C57B/6 background, candidate epitopes must be able to bind to the Class II allele, I-A^b^. Previously, it has been shown that I-A^b^ binding motifs can be predicted using algorithms based on the number of anchor residues and hydrophobicity (36–38). We measured I-A^b^ binding affinity of six candidate peptides predicted by these algorithms. The binding affinity is reflected by the amount of peptide, in nanomoles, needed to inhibit binding of a standardized radio-labeled peptide by 50% (the IC_50_) (39). Of the six peptides synthesized, only ApoB_3501–3516_ and ApoB_978–993_ bound I-A^b^ with significant affinity (Table 1, ApoB_3501–3516_ IC_50_ = 4.3 nM, ApoB_978–99_ IC_50_ = 7.3 nM). Peptides containing only parts of these sequences showed reduced binding affinities (Table 2). By comparison, the known T-cell epitopes OVA_324–337_ and MOG_38–51_ (truncated form of MOG_35–55_, i.e., myelin oligodendrocyte glycoprotein) have an IC50 of 400 nM (43) and 354 nM (44), respectively (Table 1). Based on these binding affinities, the remainder of this work was performed using ApoB_3501–3516_ and ApoB_978–993_.

**Table 2:** **I-A^b^ binding affinity of ApoB_3501–351__6_ and ApoB_978–99__3_-related peptides**.

Peptide	Sequence	Len	Pos	H-2 I-Ab (IC50 nM)
**ApoB_3501–3516_**	SFTKGNIKSSFL**SQEY**	16	3489	1169
	SSFL**SQEYSGSVANEA**	16	3497	6.8
	**SQEYSGSVANEANVY**	**15**	**3501**	**4.3**
	**SGSVANEANVY**LNSKG	16	3505	172
	**NVY**LNSKGTRSSVRLQ	16	3513	907
**ApoB_978–993_**	LFTGMNYCT**TGAYSNA**	16	969	655
	T**TGAYSNASSTESASY**	16	977	17
	**TGAYSNASSTESASY**	**15**	**978**	**7.3**
	**SSTESASY**YPLTGDTR	16	985	1258

*Sequence of ApoB_3501–3516_ and ApoB_978–993_ indicated in boldface*.

### Antigen-specific T-cell proliferative responses

Two hundred micrograms (total) of either ApoB_3501–3516_ or ApoB_978–993_ (emulsified in CFA) were injected subcutaneously into four sites in the flanks. After 10 days the draining lymph nodes were harvested, and single cell suspensions were incubated for 10 days with 100 μg/mL of either ApoB_3501–3516_ or ApoB_978–993_ (or irrelevant, MOG_35–55_) peptide for 4 days. PPD served as a positive control. Proliferation was measured by ^3^H incorporation and expressed as SI. After a single immunization of either ApoB_3501–3516_ or ApoB_978–993_ in CFA, antigen-specific T-cell proliferation was observed when relevant peptide or PPD is added, but not when irrelevant peptide is added (Table S1 in Supplementary Material).

### Immunization with ApoB_3501–3516_ and ApoB_978–993_

**Figure 1:**
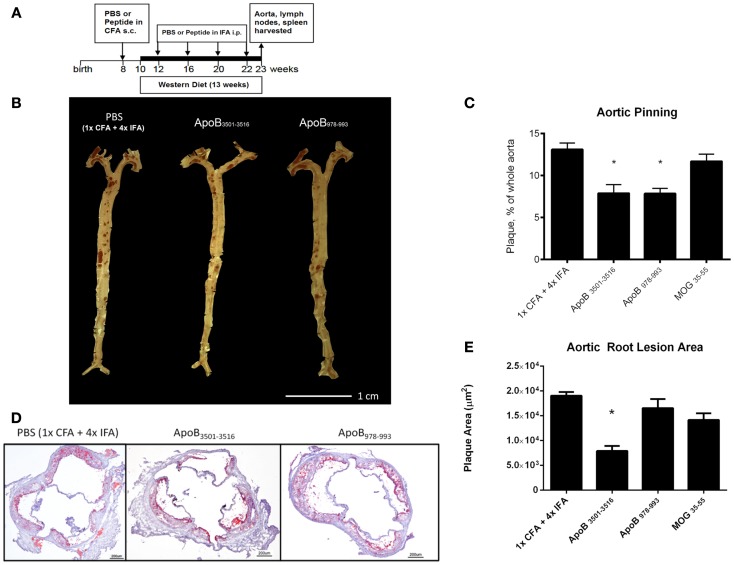
**Atherosclerosis is decreased in ApoB_3501–3516_ and ApoB_978–993_-treated mice compared to controls**. **(A)** Vaccination schedule: 8-week-old female *Apoe*^−/−^ mice were immunized once with either PBS or peptide in CFA, then boosted four more times with PBS or peptide in IFA. WD was maintained for 13 weeks. Mice were sacrificed and organs harvested at 23 weeks of age. **(B,C)** Results of aortic pinning analysis after Sudan IV staining are shown with representative photographs. *N* = 12–15 in each group, *p < 0.05 when compared to 1× CFA + 4× IFA group. **(D)** Representative aortic root staining sections after ORO staining, counter-stained with hematoxylin. **(E)** Plaque area from aortic roots stained from each group. Lesion sizes from 30 to 40 μm distal to start of the aortic valve were averaged per group. *N* = 5 in each group, *p < 0.05 when compared to 1× CFA + 1× IFA control group.

ApoB_3501–3516_ or ApoB_978–993_ were each used to vaccinate 10–14 female *Apoe*^−/−^ mice. Fifty micrograms of ApoB_3501−3516_ or ApoB_978−993_ emulsified in CFA were subcutaneously injected above the inguinal LN at 8 weeks of age. A WD was then started at 10 weeks of age. Repeated boosters with 25 μg of ApoB_3501−3516_ or ApoB_978−993_ emulsified in IFA were administered intraperitoneally at age 12, 16, 20, and 22 weeks (Figure 1A). Mice were sacrificed at age 23 weeks of age (13 weeks WD) and organs were harvested for analysis. Control immunizations with adjuvant only (1× CFA + 4× IFA) and an irrelevant peptide (MOG_35–55_) were done under identical conditions.

### Cholesterol levels

Western diet-fed *Apoe*^−/−^ mice exposed to the PBS plus 1× CFA + 4× IFA regimen had significantly decreased total plasma cholesterol from 1292 to 960 mg/dL, HDL from 246 to 168 mg/dL, and non-HDL from 1046 to 797 mg/dL, and triglycerides from 203 to 183 mg/dL compared to WD-fed *Apoe*^−/−^ mice not exposed to adjuvant. Similar observations have been reported in other studies (45). However, none of the mice immunized with ApoB_3501–3516_ or ApoB_978–993_ had plasma lipid levels different than the PBS plus 1× CFA + 4× IFA controls (Table 3). Therefore, all subsequent statistical analyses were performed without the untreated group.

**Table 3:** **Lipid profile of mice**.

	Untreated	PBS	ApoB_3501–3516_	ApoB_978–993_	MOG_35–55_
TC (mg/dL)	1292.0 ± 145.9*	960.3 ± 100.5	870.4 ± 103.6	835.2 ± 108.3	1014.7 ± 76.4
HDL (mg/dL)	245.7 ± 90.9*	167.7 ± 34.6	202.1 ± 23.8	170.3 ± 6.9	213 ± 7.5
Non-HDL (mg/dL)	1046.3 ± 105.8*	796.6 ± 119.3	663.7 ± 124.7	592.2 ± 187.1	801.7 ± 72.4
TG (mg/dL)	203.3 ± 21.0*	182.6 ± 21.9	158.1 ± 32.5	144.7 ± 37.3	142.3 ± 33.2
Weight (g)	26.8 ± 2.2	25.4 ± 1.9	26.3 ± 1.8	25.9 ± 2.1	25.3 ± 1.1

**N* = 6–7 per group, presented as mean ± SD*.

*p < 0.05 when untreated group is compared to groups exposed to adjuvant.

### Immunization with either ApoB_3501–3516_ or ApoB_978–993_ results in less atherosclerotic plaque when used in an immunization scheme using both CFA and IFA

Both ApoB_3501–3516_ and ApoB_978–993_-treated mice showed ~40% reduction in en face lesion size by Sudan IV staining of whole aortas when compared with PBS and MOG_35–55_ (Figures 1B,C) immunized mice. Aortic root lesions were also examined for plaque burden by oil red O (ORO) staining. ApoB_3501–3516_ immunized mice had >60% reduction (*p* < 0.01) in overall aortic sinus plaque development compared to 1× CFA + 4× IFA (adjuvant-only treated) mice (Figures 1D,E). ApoB_978–993_ immunized mice showed no significant reduction in aortic root plaque burden. MOG_35–55_ immunized mice showed no significant decrease. These data demonstrate that immunization with I-A^b^ restricted peptide fragments from murine ApoB-100 can reduce plaque burden in *Apoe*^−/−^ mice.

### Immunization with either ApoB_3501–3516_ or ApoB_978–993_ results in peptide-specific IgG titers

**Figure 2:**
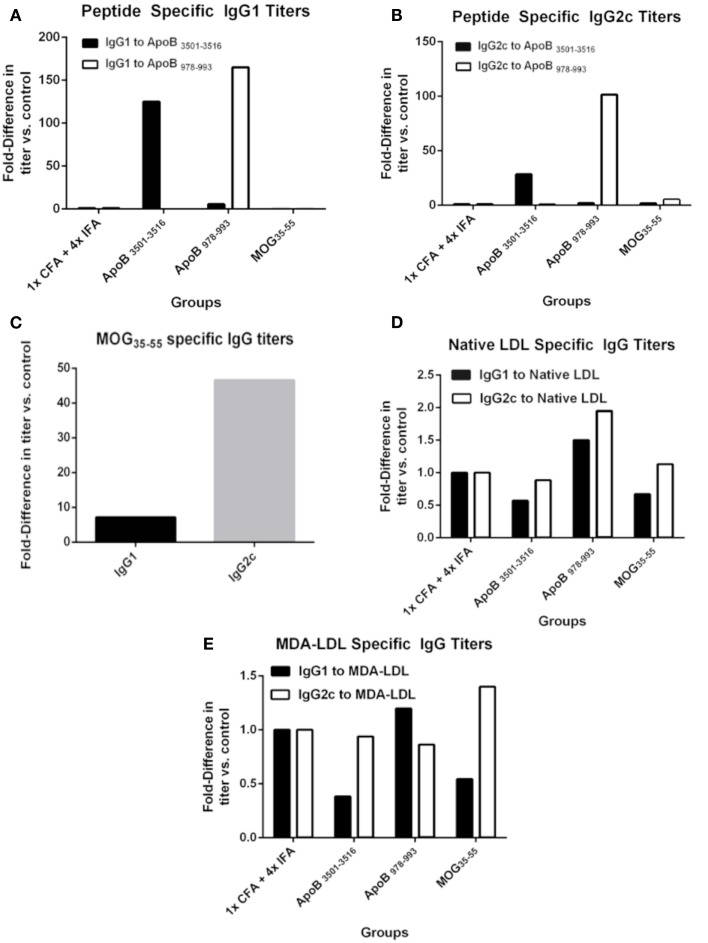
**Specific antibody titers after immunization**. Sera from 9 to 10 animals per group were pooled and formal antibody dilution curves were measured by chemiluminescent ELISA against each of the antigens. Shown here are comparative binding data at serum dilutions of 1:250 and in each case, values shown are the fold increase compared to values found with pooled sera from 1× CFA + 4× IFA group. **(A)** IgG1 titers against ApoB_3501–3516_ and ApoB_978–993_. **(B)** IgG2c titers against ApoB_3501–3516_ and ApoB_978–993_. **(C)** IgG1 and IgG2c titers against MOG35–55 in MOG_35–55_ immunized mice. IgG1 and IgG2c titers against **(D)** native (unmodified) LDL and **(E)** MDA (oxidized)-LDL.

One possible mechanism of atheroprotection is the development of protective antibodies (24). The production of IgG requires antigen-specific T-cell help and gives insight into antigen-specific T-cell activation and lineage bias. IgG1 is a marker of T-helper type 2 (T_H_2) activity and IgG2c of T-helper type 1 (T_H_1) activity in C57BL/6 mice, which do not express IgG2a (25). Pooled plasma from each group was analyzed for immunoglobulin titers by formal antibody dilution curves using chemiluminescent ELISA (Figure S1 in Supplementary Material). As shown in Figures 2A,B for the 1:250 dilution, as expected IgG responses to ApoB_3501–3516_ and ApoB_978–993_ peptides were detected in ApoB_3501–3516_ and ApoB_978–993_ immunized mice, respectively. Their responses showed complete peptide specificity, with strong responses in both the Th1 and Th2 helper T-cell compartments. Total IgG1 and IgG2c antibody levels (not antigen-specific) were similar across all groups (data not shown). MOG_35–55_ immunization produced a predominantly IgG2c response to MOG_35–55_ (Figure 2C), but no antibody titers to ApoB_3501–3516_ or ApoB_978–993_ were detected (Figures 2A,B). 1× CFA + 4× IFA immunized mice did not have detectable levels of IgG1 or IgG2c against ApoB_3501–3516_ or ApoB_978–993_ (Figures 2A,B). None of the immunized mice had elevated IgG titers against native LDL or MDA-LDL compared to adjuvant only (Figures 2D,E).

### Reduced atherosclerotic plaque burden does not correlate with an increase in the number of FoxP3-expressing cells

**Figure 3:**
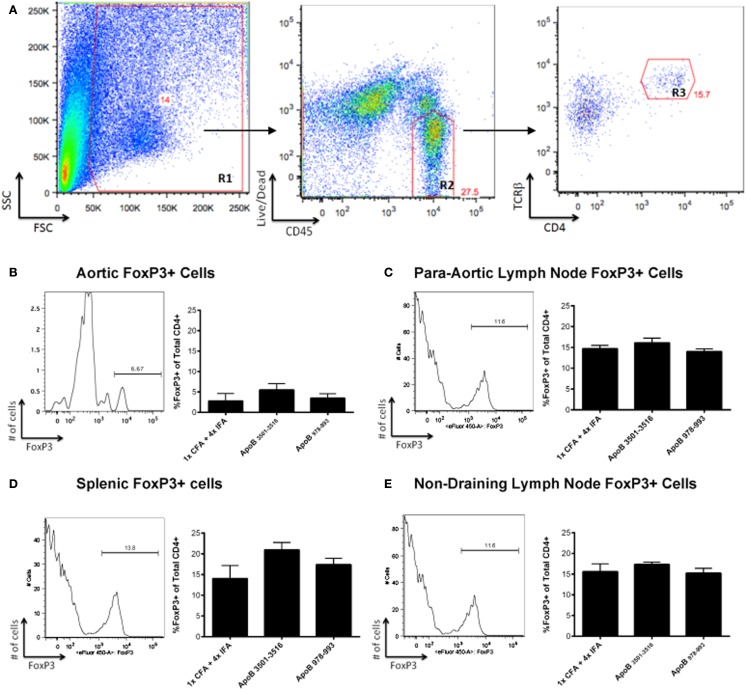
**FoxP3^+^ T-cells are not found in increased numbers in ApoB_3501–3516_ or ApoB_978–993_ immunized mice**. **(A)** The lymphocyte population was captured via forward scatter (FSC) and side scatter (SSC) gating as shown in Region 1 (R1). Live CD45^+^ cells were isolated (R2), of which TCRβ^+^/CD4^+^ cells were selected (R3) and examined for their expression of FoxP3^+^. Analysis of a representative aorta is shown. **(B)** Percentage of aortic FoxP3^+^ cells within the CD4^+^/TCRβ^+^ population. **(C)** Percentage of para-aortic lymph node FoxP3^+^ cells within the CD4^+^/TCRβ^+^ population. **(D)** Percentage of splenic FoxP3^+^ cells within the CD4^+^/TCRβ^+^ population. **(E)** Percentage of non-draining lymph nodes (pooled from inguinal, axillary, mesenteric) FoxP3^+^ cells within the CD4^+^/TCRβ^+^ population (*N* = 4–5 in each group).

Another possible mechanism of atheroprotection conveyed by immunization with ApoB_3501–3516_ or ApoB_978–993_ could be related to increased numbers of FoxP3-expressing regulatory T-cells. Whole aortas, along with spleens and lymph nodes (para-aortic, inguinal, axillary, mesenteric) were harvested from immunized mice at the time of sacrifice. There were no significant differences in FoxP3^+^ cells within the CD4^+^/TCRβ^+^ cell population in the aorta (Figures 3A,B), para-aortic lymph nodes (Figure 3C), spleens (Figure 3D), or non-draining lymph nodes (inguinal, axillary, mesenteric; Figure 3E) when ApoB_3501–3516_ and ApoB_978–993_ were compared to the 1×CFA + 4× IFA control group.

### Immunization with ApoB_3501–3516_ or ApoB_978–993_ increases mRNA expression of the atheroprotective cytokine, IL-10

**Figure 4:**
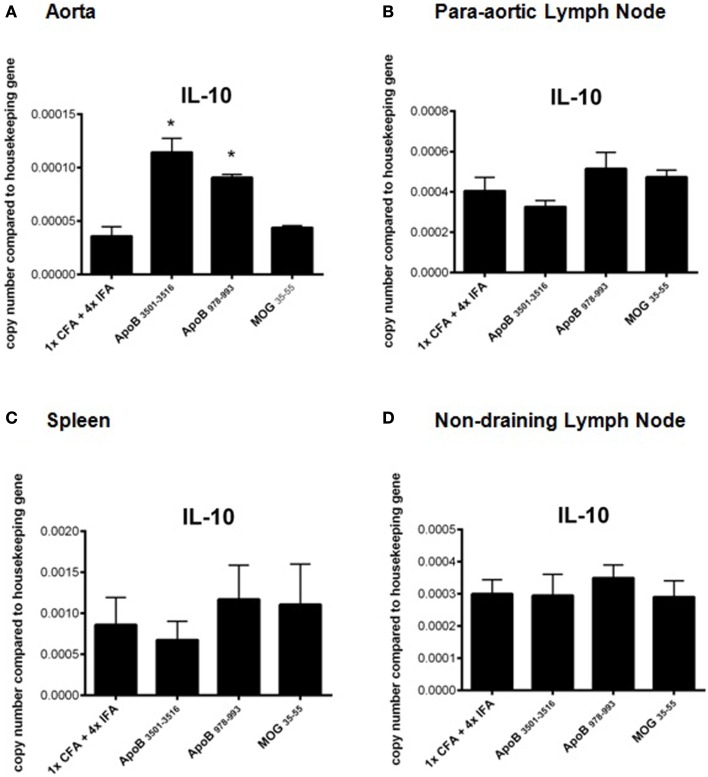
**Real time RT-PCR analysis of IL-10 mRNA expression**. Organs were harvested and immediately placed into RNA stabilization reagent, and frozen at −80^°^C. IL-10 mRNA expression from **(A)** aortas, **(B)** para-aortic lymph nodes, **(C)** spleens, and **(D)** non-draining lymph nodes are shown. *p < 0.05 compared to 1× CFA + 4× IFA. *N* = 3–5 in each group.

RT-PCR analysis of spleens, lymph nodes, and aortas of mice from each group were analyzed for mRNA expression levels of the T_H_1 cytokines IFNγ, TNFα, and the T_H_1 transcription factor Tbx21 (Tbet), the T_H_2 cytokines IL-4, IL-10, and the T_H_2 transcription factor GATA3, the T_H_17 cytokine IL-17A, and the T_H_17 transcription factor RORγT, and the regulatory T-cell transcription factor FoxP3. No significant differences were found except in aortas of ApoB_3501–3516_ and ApoB_978–993_ immunized mice where a significant increase in IL-10 mRNA expression was noted (*p* < 0.05) compared to 1× CFA + 4× IFA treated and MOG_35–55_ immunized control mice (Figures 4A–D). There was no significant difference in mRNA expression of any other cytokine (IFNγ, TNFα, IL-4, or IL-17A) or transcription factor (Tbet, GATA3, RORγT, or FoxP3) examined between groups in any organ (data not shown).

## Discussion

Our results show that MHC Class II restricted CD4^+^ T-cell peptides from the ApoB-100 are effective at reducing atherosclerotic plaque burden in *Apoe*^−/−^ mice. ApoB_3501–3516_ or ApoB_978–993_ are high affinity binders for MHC Class II (I-A^b^). This is the first publication to identify such peptides within an atherosclerosis-relevant protein (ApoB-100).

T-cell responses to vaccination with either (ox)LDL or peptide epitopes from ApoB-100 have become an area of great interest because they may enable the development of a vaccine for clinical use. Recent reports have suggested that immunization with an epitope from ApoB-100 that binds to serum antibodies, P210, results in atheroprotection via an increase in T_REGS_ (32, 33), but the mechanism by which a B-cell epitope could induce T_REGS_ was not elucidated. It is not known whether these T_REGS_ are causally related to the atheroprotection that is observed. In fact, P210 does not bind to I-A^b^ (34), suggesting that any actions immunizing with P210 might have are highly unlikely to be related to CD4^+^ helper T-cells. One publication has instead suggested that the observed atheroprotection is a result of enhanced CD8^+^ cytotoxic T-cell activity against dendritic cells leading to a decreased number of CD11c^+^ cells within the aorta, and thus reduced atherosclerosis (46). These researchers demonstrated that adoptive transfer of P210 primed CD8^+^ T-cells recapitulated the atheroprotective response in naïve mice. These contrasting views of how immunization may result in atheroprotection highlight the need for systematic studies into MHC-restricted peptide vaccinations.

Mice immunized with ApoB_3501–3516_ or ApoB_978–993_ in the context of CFA and IFA show increased IL-10 mRNA expression levels in the aortas, significantly above control mice. Since this increased expression is not associated with an increased in the percent of aortic FoxP3^+^ regulatory T-cells, we speculate that the IL-10 mRNA may be derived from FoxP3^−^ Tr1 cells (47, 48) (i.e., one subset of inducible T_REGS_) or from myeloid cells (49). While both IFNγ and IL-17A have been implicated as pro-atherosclerotic cytokines, no changes in mRNA expression of either cytokine was observed in the aortas, lymph nodes, or spleens of immunized mice compared to controls.

We did not detect IgG1 or IgG2c antibody titers to ApoB_3501–3516_ or ApoB_978–993_ in non-immunized mice. This is despite highly elevated plasma cholesterol levels in *Apoe*^−/−^ mice (50). One possibility is that ApoB_3501–3516_ and ApoB_978–993_ may not be naturally processed products of APCs. Another possibility is that the endogenous forms of ApoB_3501–3516_ and ApoB_978–993_ are not presented efficiently or in high enough quantities by APCs. Further investigations into the mechanism of atheroprotection will provide new targets for therapy and prevention of atherosclerosis. It is our hope that these two peptides, and future peptides that can be discovered by the described immunologic methods, will lead to a new frontier in atherosclerosis research and ultimately provide a treatment for this worldwide epidemic.

## Conflict of Interest Statement

The authors declare that the research was conducted in the absence of any commercial or financial relationships that could be construed as a potential conflict of interest.

## Supplementary Material

The Supplementary Material for this article can be found online at http://www.frontiersin.org/Journal/10.3389/fimmu.2013.00493/abstract

Figure S1 **Specific antibody dilution curves**. Antibody specific titers were measured by formal antibody dilution curves using chemiluminescent ELISA. Dilutions of 1:50 (when possible), 1:250, 1:2500 and 1:6250 were performed. *Baseline* group (blue line) represents pooled serum from two female *Apoe^−/−^* mice at 8 weeks of age on chow diet, without immunization. The *untreated* group (green line) represents 9–10 female *Apoe^−/−^* mice fed western diet for 13 weeks, starting at 10 weeks of age but without any immunizations. Data are expressed as relative light units counted per 100 ms (RLU/100 ms). **(A)** IgG1 titers to ApoB_3501–3516_, ApoB_978–993_, and MOG_35–55_. **(B)** IgG2c titers to ApoB_3501–3516_, ApoB_978–993_, and MOG_35–55_. **(C)** IgG1 and IgG2c titers to native (unmodified) LDL. **(D)** IgG1 and IgG2c titers to MDA(oxidized)-LDL.

Table S1 **Antigen-specific T-cell proliferation**. Mice were immunized with either ApoB_3501–3516_, ApoB_978–993_, PPD (positive control), or MOG_35–55_ (negative control). Draining lymph node cells were harvested 10 days later and incubated with the relevant peptide, PPD or MOG_35–55_. Proliferative responses were measured by ^3^H incorporation and expressed as SI. We show here that there are antigen-specific T-cell responses to both ApoB_3501–3516_ and ApoB_978–993_.
