# Supplementary material for: Atheroprotective Vaccination with MHC-II Restricted Peptides from ApoB-100
Source: Front Immunol. 2013 Dec 27;4:493. doi: 10.3389/fimmu.2013.00493 (PMC3873602; doi:10.3389/fimmu.2013.00493)
Supplement: Figure S1 — Specific antibody dilution curves. Antibody specific titers were measured by formal antibody dilution curves using chemiluminescent ELISA. Dilutions of 1:50 (when possible), 1:250, 1:2500 and 1:6250 were performed. Baseline group (blue line) represents pooled serum from two female Apoe−/− mice at 8 weeks of age on chow diet, without immunization. The untreated group (green line) represents 9–10 female Apoe−/− mice fed western diet for 13 weeks, starting at 10 weeks of age but without any immunizations. Data are expressed as relative light units counted per 100 ms (RLU/100 ms). (A) IgG1 titers to ApoB3501–3516, ApoB978–993, and MOG35–55. (B) IgG2c titers to ApoB3501–3516, ApoB978–993, and MOG35–55. (C) IgG1 and IgG2c titers to native (unmodified) LDL. (D) IgG1 and IgG2c titers to MDA(oxidized)-LDL. [file DataSheet1.ZIP › Presentation 2.PDF]

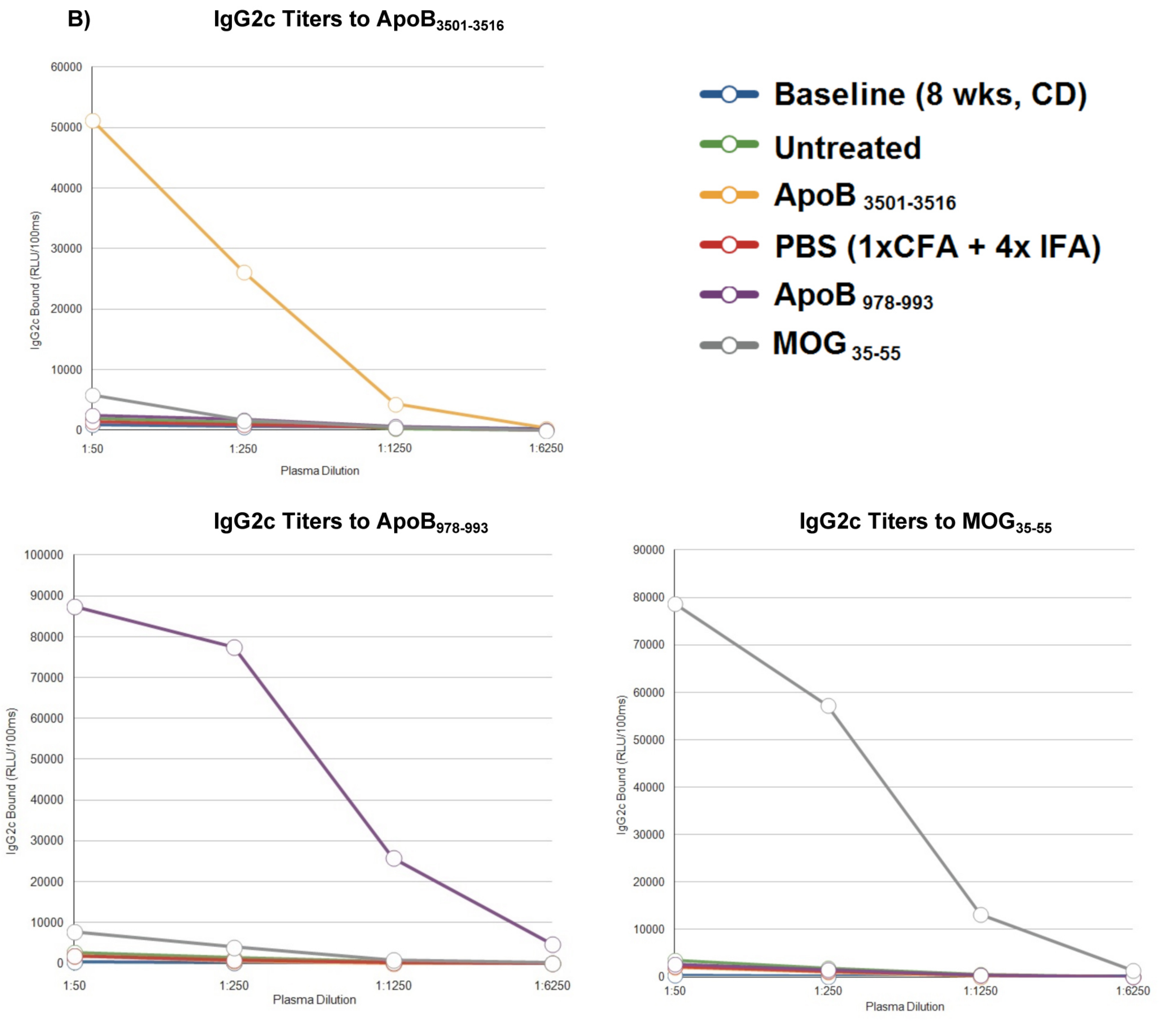
B)
IgG2c Titers to ApoB3501-3516
60000
50000
40000
30000
20000
10000
0
IgG2c Bound (RLU/100ms)
1:50
1:250
1:1250
1:6250
Plasma Dilution
Baseline (8 wks, CD)
Untreated
ApoB 3501-3516
PBS (1xCFA + 4x IFA)
ApoB 978-993
MOG 35-55
IgG2c Titers to ApoB978-993
100000
90000
80000
70000
60000
50000
40000
30000
20000
10000
0
IgG2c Bound (RLU/100ms)
1:50
1:250
1:1250
1:6250
Plasma Dilution
IgG2c Titers to MOG35-55
90000
80000
70000
60000
50000
40000
30000
20000
10000
0
IgG2c Bound (RLU/100ms)
1:50
1:250
1:1250
1:6250
Plasma Dilution
